# Potential Benefits of Flunarizine in Patients with Sudden Sensorineural Hearing Loss with Incomplete Recovery Following Conventional Steroid Treatment: A Retrospective Analysis

**DOI:** 10.3390/medicina61040769

**Published:** 2025-04-21

**Authors:** Young-Soo Chang, Jeong Hwan Choi

**Affiliations:** Department of Otorhinolaryngology-Head and Neck Surgery, Sanggye Paik Hospital, College of Medicine, Inje University, Seoul 01757, Republic of Korea; choijh92@paik.ac.kr

**Keywords:** sudden sensorineural hearing loss, steroid, flunarizine, migraine, intratympanic

## Abstract

*Objectives*: This study assessed the potential benefits of supplemental flunarizine administration in patients with sudden sensorineural hearing loss (SSNHL) experiencing incomplete recovery with conventional steroid treatment. *Methods*: Thirty-nine SSNHL patients, unresponsive to conventional steroid therapy, received either oral ginkgo biloba (control group, *n* = 24) or a combination of oral ginkgo biloba and flunarizine (treatment group, *n* = 15). Pure-tone average (PTA) evaluation at 0.5, 1, 2, and 4 kHz was conducted upon each patient’s visits. A change of ≥10 dB was considered ‘significant hearing gain’. *Results*: The treatment group showed a higher rate of hearing gain ≥10 dB (46.7%) compared to the control group (12.5%) (*p* = 0.03). Additional treatment with flunarizine was associated with greater improvement in hearing thresholds compared with ginkgo biloba alone (*p* = 0.004), suggesting a potential therapeutic benefit. *Conclusion*: This retrospective study suggests that flunarizine may provide additional benefit in patients with SSNHL experiencing incomplete recovery following conventional steroid treatment. These findings are preliminary and require validation in larger, controlled studies.

## 1. Introduction

Sudden sensorineural hearing loss (SSNHL), defined as a hearing loss of at least 30 dB at three sequential frequencies in a standard pure-tone audiogram occurring over 72 h [1], is an otologic emergency because successful recovery depends on prompt treatment [2]. In addition to the time from onset to the beginning of treatment, factors such as age, initial hearing levels, and the presence of vertigo are known prognosticators of hearing recovery [3].

Corticosteroids are considered to be an effective initial treatment for SSNHL [4]. The anti-inflammatory effects of corticosteroids, as shown in lab studies, are believed to be the key mechanism that allows corticosteroids to be effective in treating SSNHL. Corticosteroids can be delivered via the oral and/or intratympanic (IT) routes. For patients with SSNHL who fail to respond to the initial treatment, IT steroid injection (ITSI) or hyperbaric oxygen therapy have been proposed as salvage therapies to obtain additional hearing recovery [5,6].

Despite these salvage therapies, a large proportion of patients with SSNHL continue to experience limited hearing recovery. Because it has been reported that corticosteroids show effectiveness within 2 to 4 weeks of the onset of SSNHL [7,8], patients with incomplete hearing recovery often do not receive any additional treatment and continue to endure functional decline and poor quality of life [9].

Recently, the role of adjuvant migraine treatment in SSNHL patients has been suggested [10,11]. When adjuvant migraine treatment was added to conventional oral and IT steroid therapies, outcomes improved. In addition, SSNHL patients seeking medical attention at least 6 weeks after initial symptomatic onset showed significant improvements in their speech-recognition-threshold and word-recognition scores. This may be associated with the possible etiologic role of migraine, which is suggested to be partially vascular in nature, thus resulting in the higher prevalence of migraines among SSNHL patients [12,13].

Flunarizine, commonly used to treat chronic migraines, is part of a family of calcium channel blockers which have selective effects on cerebrovascular circulation. Its mechanism of action involves reducing vascular smooth muscle spasms. Ménière’s disease, vertigo, and tinnitus are the indications for these drugs in the otologic field [14]. This retrospective study is aimed at analyzing the potential benefits of flunarizine treatment in patients with SSNHL experiencing incomplete recovery following conventional steroid treatment.

## 2. Materials and Methods

### 2.1. Participants

This study received approval from the Institutional Review Board (IRB No. 2022-07-007). We conducted a survey of individuals experiencing sudden hearing loss in one ear between January 2020 and June 2023. The inclusion criteria in this study were as follows: (i) onset of hearing loss occurred within the last 72 h with ≥30 dB hearing loss at three consecutive frequencies and confirmed with pure-tone audiometry; (ii) those who underwent oral methylprednisolone (MPD) and sequential ITSI but did not achieve complete hearing recovery in 1 month; and (iii) patients who were administered oral ginkgo biloba at a dose of 80 mg twice a day (Ginexin-F, SK Pharma Co., Ltd., Seoul, Republic of Korea) for a 3-month period.

The exclusion criteria were as follows: (i) previous or current history of otologic disease including otitis media, myringitis, traumatic tympanic perforation, vestibular schwannoma, or Ménière’s disease; (ii) any medication related to SSNHL (such as systemic or intratympanic steroids, antivirals agents, or vasodilators) administered prior to the patient’s visit to our clinics; (iii) patients who declined sequential ITSI administration; and (iv) patients with any history of essential tremor.

### 2.2. Treatment Protocol

All patients were prescribed a standardized protocol, which included oral MPD at a dose of 48 mg daily for 7 days, with subsequent sequential tapering for 4 days. The daily doses of MPD were given at the same time every day (9 a.m.). Oral ginkgo biloba (80 mg, twice daily) was co-administered from the beginning of treatment.

When complete hearing recovery was not achieved within 5 days of initiating MPD, intratympanic steroid injections (ITSI) were initiated. A total of four ITSI sessions were administered at two-day intervals. As a result, ITSI treatment extended beyond the systemic MPD schedule, and during this period, patients received only oral ginkgo biloba in conjunction with ITSI.

IT treatment was performed using the following method; patients were placed in a sitting position tilted backward 30°, and their head was tilted at 40–45° to the healthy side. Local anesthesia was then administered. Then, a 26-gauge spinal needle was introduced under microscopy to the anterior–superior tympanic membrane. A minimum of 0.3 mL of the steroid solution (dexamethasone, 5 mg/mL, Dexa-S; Il-Sung Pharmaceutical, Seoul, Republic of Korea) was gently perfused into the middle ear. After the injection was performed, patients were advised to avoid moving their head, speaking, or swallowing for 30 min.

If complete hearing recovery was not achieved within one month of the initial visit, patients underwent routine audiometric follow-ups while continuing oral ginkgo biloba for up to 3 months after the onset of symptoms.

Patients were categorized into two groups based on additional treatment modalities: the control group, who received only oral ginkgo biloba, and the treatment group, who received both oral ginkgo biloba and flunarizine. Starting from September 2021, additional flunarizine treatment for incomplete recovery from SSNHL was performed. In the treatment group, administration of flunarizine was initiated at the one-month follow-up visit in patients who exhibited insufficient hearing recovery by that time. Since flunarizine treatment was introduced as a routine practice only after September 2021, group allocation was determined by the treatment period and not pre-specified. Consequently, patients were not informed of their group assignment and were unaware whether they were part of the flunarizine treatment group, resulting in an inherent level of blinding at the patient level. A daily dose of 5 mg of oral flunarizine was prescribed for 2 months. Monthly monitoring included close interviews and evaluation of any changes in the patient’s general condition, mood, and movement, to detect any potential side effects of flunarizine administration.

### 2.3. Outcome Evaluation

Pure-tone audiometry was conducted on all patients upon their initial visit and again 2 weeks, 4 weeks, and 12 weeks after commencing the treatment protocol. Pure-tone air conduction threshold audiometry was conducted using a Natus Inc. (Taastrup, Denmark) Madsen Astera^2^ audiometer with TDH-39P headphones. The audiometry recorded pure-tone thresholds at frequencies of 0.25, 0.5, 1.0, 2.0, 3.0, 4.0, and 8.0 kHz. For thresholds that could not be tested due to equipment limitations, a ceiling of 100 dB HL was set for the present study.

The pure-tone average (PTA) was calculated based on the mean thresholds at four frequencies (0.5, 1, 2, and 4 kHz). Additionally, we determined the hearing gain using the following formula: hearing gain (dB HL) = (one-month PTA) − (three-months PTA). A change of ≥10 dB HL was considered ‘significant hearing gain’ in the present study. This PTA-based definition was used solely to assess overall hearing improvement. For frequency-specific comparisons between groups, hearing gains were analyzed independently at each frequency using statistical methods. Thus, statistical significance at individual frequencies was determined through intergroup comparisons of mean changes at each frequency.

### 2.4. Statistical Analyses

Quantitative variables were summarized using the mean (and standard deviation) and/or median. Categorical variables were presented as frequencies. The frequencies at which significant hearing gain occurred between the two groups were evaluated using Fisher’s exact test. Multivariable linear regression analysis was conducted to evaluate the factors associated with hearing gain after the administration of MPD and sequential ITSI in cases with incomplete hearing recovery. Initial PTA, treatment delay, treatment modality, and diabetes were considered as possible associated factors. In analyzing treatment modality as a potential factor, the control group was considered as a reference. A two-sided *p* value < 0.05 was considered as statistically significant. All data were analyzed with SPSS software, version 20.0 (SPSS, Chicago, IL, USA).

## 3. Results

Thirty-nine patients were enrolled and the data were reviewed. The mean age of the patients was 60.2 ± 13.5 years (range, 19–89 years). There were 13 males (33.3%) and 26 females (66.7%). Common medical histories included diabetes (5, 12.8%) and hypertension (12, 30.8%). On the initial visit, there were complaints of tinnitus (23, 59%) and dizziness (11, 28.2%). After the conventional treatment, 24 patients received oral ginkgo biloba only (control group) and 15 patients received both oral ginkgo biloba and flunarizine (treatment group). There were no significant differences in the baseline characteristics between the two groups. The initial PTAs in the control and treatment groups were 65.1 ± 27.5 dB HL and 59.5 ± 23.1 dB HL, respectively. The mean PTAs of the two groups at 2 weeks and 4 weeks had no significant differences. Detailed demographic characteristics of the patients are shown in Table 1.

The number of patients with significant hearing gain at 12 weeks was as follows: 3 (12.5%) in the control group and 7 (46.7%) in the treatment group (Table 2, *p* = 0.03, Fisher’s exact test). A multivariate linear regression analysis was performed, considering hearing gain as a dependent, continuous variable (Table 3, adjusted *R*^2^ = 0.17). Patients who received additional treatment showed a significant improvement in the degree of hearing gain compared with patients from the control group (unstandardized regression coefficient = 6.44, *p* = 0.004). Although the result (*p* = 0.06) did not reach the threshold for significance, individuals with diabetes showed a negative association with hearing gain (*B* = −0.58).

**Figure 1:**
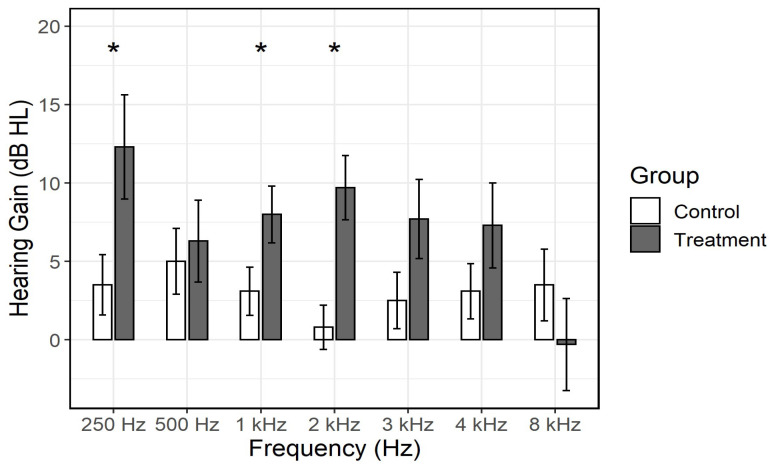
Comparison of mean hearing gain between the control group and the flunarizine-treated group across different frequencies. Asterisks (*) indicate statistically significant differences between groups (*p* < 0.05). Error bars represent standard error of the mean.

A comparison of the hearing gain at different frequencies between the two groups is presented in Figure 1. The group receiving additional flunarizine treatment showed a statistically significant hearing gain compared to the control group at the following frequencies: 250 Hz, 1 kHz, and 2 kHz (*p* = 0.03, *p* = 0.048, and *p* = 0.001, respectively).

Complications with flunarizine administration were minimal in this study. None of the patients who received flunarizine showed any tiredness, drowsiness, weight gain, low mood, or tremors.

## 4. Discussion

In this retrospective study focusing on SSNHL patients with incomplete recovery following conventional oral and intratympanic steroid treatment, our results indicate that additional administration of flunarizine led to clinical benefits, resulting in an additional hearing recovery of 6.44 dB (the regression coefficient in our study). Notably, 46.7% of the patients in the flunarizine treatment group showed a significant hearing gain, defined as a change of ≥10 dB following the administration of additional flunarizine 1 month after the initial treatment. A previous study reported delayed recovery occurring later than 1 month after discharge in 9.9% of 121 patients who were treated with combination therapy including oral corticosteroids over a period of three months [15]. Our data showed a higher rate of delayed recovery (46.7%) in the flunarizine treatment group compared to the previous study over a period of 3 months, while the control group showed a similar rate of delayed recovery (12.5%). This suggests that additional flunarizine administration may offer benefits in terms of further hearing improvement in SSNHL patients with incomplete recovery, compared to conventional treatment. The observed improvements in this present study align with those documented in a previous report which found that SSNHL patients treated with migraine medications may experience at least partial reversibility even after the traditional 30-day post-onset window [11]. These findings contribute to the growing understanding of potential therapeutic avenues for SSNHL patients with incomplete recovery and underscore the importance of exploring alternative treatment modalities beyond the standard approaches.

A previous investigation reported the effects of supplementing oral and IT steroids with migraine medications as the primary treatment for SSNHL [10]. They prescribed a combination of nortriptyline and topiramate at the initial visit for patients with SSNHL. The study demonstrated that supplementing oral and IT steroids with migraine medications led to significant hearing recovery at low frequencies (250, 500, and 1000 Hz). This result is similar to the results we obtained in our specific frequency analysis, which showed hearing gains with additional flunarizine administration at 250 Hz, 1 kHz, and 2 kHz. Although the initiation timing of migraine medication varied between the studies, these consistent results emphasize the importance of further investigating the effects and mechanisms of migraine medication in the context of SSNHL. These parallel findings across different studies may offer valuable insights into potential therapeutic strategies for patients with SSNHL, highlighting the potential benefits of integrating migraine medications into treatment protocols.

A recent longitudinal study reported that 27% of SSNHL patients experienced persistent hearing loss after 1 month, despite seeking medical attention within 10 days of symptom onset and receiving steroid therapy [16]. The efficacy of any additional treatment on refractory SSNHL following conventional steroid treatment has not been fully elucidated and patients with incomplete recovery are often limited to follow-up investigations without additional treatment. Therefore, it is important to examine additional treatments that might be effective alongside conventional steroid treatments. Our study has produced a number of significant results. Our data showed significant hearing improvement 30 days after the initial visit (as measured by PTA) with the additional administration of flunarizine following conventional steroid treatment. This study offers a clear and straightforward understanding of the results, with the most significant result being derived from the comparison with the control group. These results can contribute to a broader exploration of the therapeutic options for individuals facing refractory SSNHL, offering insights that may inform future treatment strategies.

Numerous studies have identified an association between SSNHL and the trigeminal innervation of the cochlea vasculature, which is similar to the pathogenesis of migraine headaches [17,18]. Clinical evidence suggests that patients with migraines have an increased risk of developing SSNHL, constituting a disproportionately large portion of SSNHL cases [13]. Kim et al. reported that the adjusted hazard ratio of migraine for SSNHL was 1.34 (95% CI 1.19–1.50). Furthermore, various otologic manifestations, such as hyperacusis, prolonged aural fullness, vertigo, and Ménière’s disease, have demonstrated responsiveness to migraine medications [19,20,21]. The link between SSNHL and migraines may be due to the underlying nature of migraine, a vasospasm in the cochlear vasculature [22]. Migraine medications may thus provide therapeutic benefits for both migraines and SSNHL by altering vascular flow and addressing a potentially similar underlying pathophysiology [10].

Flunarizine is a calcium channel blocker and has been demonstrated as an effective therapy for Ménière’s disease, vertigo, and tinnitus [14]. Compared to other migraine prophylactics, such as verapamil, amitriptyline, and valproate, flunarizine does not need dosage escalation for symptomatic improvement of migraines. It should be kept in mind that several of the side effects, such as tiredness, drowsiness, weight gain, and low mood, may be caused by prolonged flunarizine administration. Particular attention should be given to the potential risk of flunarizine-related movement disorders when clinicians prescribe flunarizine as a supplemental therapy. If a patient experiences any behavioral problems after taking flunarizine, the flunarizine should be discontinued as soon as possible to ensure prompt recovery from the movement disorders [23]. The risk of flunarizine-related movement disorders is increased in patients with higher dose exposure, older age, and with a history of essential tremor or cardiovascular disease. In the present study, we only prescribed flunarizine for a period of 2 months, with a single dose (5 mg) per day, and we excluded patients with any history of essential tremor, to minimize any possible side effects. However, it is important to highlight that the maximum daily dosage of flunarizine for patients under the age of 65 is 10 mg. This indicates the need for further research to determine the appropriate dosage for efficacy of flunarizine treatment for SSNHL patients.

According to the 2019 clinical practice guideline [4], systemic corticosteroids may be offered as an initial treatment option for patients with SSNHL within 2 weeks of symptom onset (KAS 8, “option”). Furthermore, ITSI are recommended as a salvage therapy in patients who exhibit incomplete recovery within 2 to 6 weeks (KAS 10, “recommendation”). In contrast to these flexible recommendations, the present study employed a more structured and standardized treatment protocol, initiating systemic MPD promptly upon diagnosis, followed by ITSI beginning on day 6 in patients without sufficient recovery. This reflects a more proactive approach to combined steroid therapy compared to the guideline, where initial ITSI or combination therapy is not emphasized.

Additionally, the long-term use of oral ginkgo biloba for up to three months, as well as the delayed introduction of flunarizine in selected patients with insufficient recovery at one month, are not addressed in the current guideline. While these adjunctive treatments are not part of the standard guideline recommendations, their inclusion may reflect efforts to enhance long-term auditory outcomes and provide alternative therapeutic options for patients with poor early recovery. Further research is warranted to evaluate their efficacy in larger, controlled cohorts.

This study has several limitations. First, the sample size was relatively small, primarily due to the strict application of the inclusion and exclusion criteria. This should be kept in mind when interpreting the outcomes, as the findings are based on a limited retrospective case series involving patients with incomplete recovery following conventional steroid treatment. Second, to ensure a relatively homogeneous study group, we only included patients who had completed the same treatment protocol consisting of oral MPD followed by sequential ITSI. Patients who did not consent to ITSI, despite incomplete hearing recovery within 1 month, were excluded from the analysis. As a result, there is a potential for selection bias, and the findings of this study may not be generalizable to patients who refuse ITSI or follow different therapeutic protocols. Third, a placebo control group was not included, as all patients received oral ginkgo biloba in accordance with standard local practice. To avoid withholding treatment, the study evaluated the additive effect of flunarizine rather than comparing it directly to placebo, which limits interpretation of its isolated efficacy. In addition, the potential influence of placebo effects should be acknowledged. Idiopathic SSNHL is known to have a high rate of spontaneous recovery, and placebo responses may have contributed to the observed hearing improvements, particularly in patients receiving invasive interventions such as ITSI. Furthermore, differences in treatment modality—oral medications versus intratympanic injections—may introduce psychological or contextual biases that influence perceived outcomes. These factors underscore the importance of future randomized, double-blind, placebo-controlled studies, to more accurately determine therapeutic efficacy. Finally, this study did not compare the effectiveness of different prophylactic migraine medications. Given the complex nature of migraine, individualized prescriptions may maximize the benefit of additional migraine-targeted treatments in SSNHL patients. This issue could be further addressed in a large-scale, controlled study to better understand the underlying mechanisms and treatment effects.

## 5. Conclusions

In summary, our findings suggest a potential benefit of additional flunarizine administration in patients with SSNHL who exhibit incomplete recovery following conventional steroid treatment. Given the retrospective nature and limited sample size, these results should be interpreted with caution. Further evaluation in larger, randomized controlled trials is necessary to confirm these observations.

## Figures and Tables

**Table 1:** Baseline characteristics.

	Control (*n* = 24)	Treatment (*n* = 15)	*p* Value
Sex			
Male	7	6	0.73
Female	17	9	
Age	61.5 ± 15.0	57.9 ± 10.7	0.39
Treatment delay (days)	6.0 ± 7.7	5.6 ± 5.7	0.85
Diabetes			
No	23	11	0.06
Yes	1	4	
Hypertension			
No	17	10	1
Yes	7	5	
Tinnitus			
No	11	5	0.66
Yes	13	10	
Dizziness			
No	18	10	0.72
Yes	6	5	
Laterality			
Right	15	7	0.52
Left	9	8	
Initial PTA (dB HL)	65.1 ± 27.5	59.5 ± 23.1	0.50
PTA at 2 weeks (dB HL)	59.1 ± 26.3	51.3 ± 22.6	0.35
PTA at 4 weeks (dB HL)	53.8 ± 22.8	51.8 ± 20.7	0.78
Hearing threshold at the initial visit			
250 Hz	56.2 ± 32.8	56.3 ± 26.1	0.99
500 Hz	62.7 ± 32.5	56.7 ± 27.6	0.54
1 kHz	65.0 ± 30.5	58.7 ± 25.4	0.89
2 kHz	63.3 ± 29.5	55.0 ± 25.6	0.36
3 kHz	65.0 ± 27.7	65.3 ± 20.7	0.97
4 kHz	69.2 ± 27.4	67.7 ± 21.2	0.85
8 kHz	76.7 ± 23.8	67.0 ± 26.5	0.26

**Table 2:** Distribution of significant hearing gain in audiograms taken at 3 months according to presence or absence of flunarizine treatment.

		Control	Treatment	*p* Value
Significant Hearing Gain	Yes	3	7	0.03 *
	No	21	8	
Total		24	15	

*: *p* < 0.05.

**Table 3:** Multivariable analysis of factors potentially associated with hearing gain.

	B	SE	*p* Value	95% CI
Flunarizine	6.44	2.06	0.004 *	2.25–10.63
Diabetes	−5.81	3.00	0.06	−11.91–0.29
Treatment Delay	0.03	0.15	0.87	−0.27–0.32
Age	−0.02	0.08	0.78	−0.18–0.13
Initial PTA (dB HL)	0.07	0.04	0.10	−0.01–0.15
Contrast	−5.92	5.72	0.31	−17.56–5.73

*: *p* < 0.05; B: unstandardized regression coefficient; SE: standard error; CI: confidence interval; PTA: pure-tone average at 0.5, 1, 2, and 4 kHz.

## Data Availability

All data and materials used and analyzed during the course of this study are available from the corresponding author upon reasonable request.

## Abbreviations

The following abbreviations are used in this manuscript:

SSNHL	Sudden sensorineural hearing loss
ITSI	Intratympanic steroid injection
MPD	Methylprednisolone
PTA	Pure-tone average
